# Patient-specific quality assurance strategies for synthetic computed tomography in magnetic resonance-only radiotherapy of the abdomen

**DOI:** 10.1016/j.phro.2023.100464

**Published:** 2023-06-26

**Authors:** Riccardo Dal Bello, Mariia Lapaeva, Agustina La Greca Saint-Esteven, Philipp Wallimann, Manuel Günther, Ender Konukoglu, Nicolaus Andratschke, Matthias Guckenberger, Stephanie Tanadini-Lang

**Affiliations:** a Department of Radiation Oncology, University Hospital Zurich and University of Zurich, Zurich, Switzerland; b Artificial Intelligence and Machine Learning Group, Department of Informatics, University of Zurich, Zurich, Switzerland; c Computer Vision Laboratory, ETH Zurich, Zurich, Switzerland

**Keywords:** MR-guided radiotherapy, synthetic CT, Neural network, Quality assurance, PSQA, MR-Linac

## Abstract

**Background and purpose:**

The superior tissue contrast of magnetic resonance (MR) compared to computed tomography (CT) led to an increasing interest towards MR-only radiotherapy. For the latter, the dose calculation should be performed on a synthetic CT (sCT). Patient-specific quality assurance (PSQA) methods have not been established yet and this study aimed to assess several software-based solutions.

**Materials and methods:**

A retrospective study was performed on 20 patients treated at an MR-Linac, which were selected to evenly cover four subcategories: (i) standard, (ii) air pockets, (iii) lung and (iv) implant cases. The neural network (NN) CycleGAN was adopted to generate a reference sCT, which was then compared to four PSQA methods: (A) water override of body, (B) five tissue classes with bulk densities, (C) sCT generated by a separate NN (pix2pix) and (D) deformed CT.

**Results:**

The evaluation of the dose endpoints demonstrated that while all methods A-D provided statistically equivalent results (p = 0.05) within the 2% level for the standard cases (i), only the methods C-D guaranteed the same result over the whole cohort. The bulk densities override was shown to be a valuable method in absence of lung tissue within the beam path.

**Conclusion:**

The observations of this study suggested that the use of an additional sCT generated by a separate NN was an appropriate tool to perform PSQA of a sCT in an MR-only workflow at an MR-Linac. The time and dose endpoints requirements were respected, namely within 10 min and 2%.

## Highlights

• The use of a separate neural network provided dose equivalence within 2%
• The time burden was limited to less than 10 min.
• The method was shown to be applicable in presence of air pockets, lung or implants.

## 1 Introduction

Magnetic resonance (MR) provides superior soft tissue contrast compared to computed tomography (CT) and its integration in radiation therapy (RT) treatment planning has become the standard of care for several treatment sites [1], [2]. The clinical introduction of the new generation hybrid devices coupling linear accelerators (Linac) with MR scanners (MR-Linac) has broadened even further the benefits that MR can provide to the RT workflow [3], [4], [5]. These include, among others, patient positioning without radiation exposure, daily plan adaptation based on inter-fraction anatomical changes and radiation gating based on intra-fraction internal organ motion [6], [7], [8], [9]. Given that MR has become the primary imaging modality for MR-Linac treatments, efforts have been made to simplify the treatment planning workflow aiming to eliminate the CT simulation and to transition towards MR-only radiotherapy [10]. In this context, the generation of a synthetic CT (sCT) from the MR data is a requirement to obtain the electron density map and perform dose calculation. The dose deviations with respect to the planning CT should be limited below 2% for clinical applicability [11].

Several methods have been proposed for the sCT generation. The initial investigations were based on tissue segmentation and atlas-based approaches, which were limited by the long computation times [12]. The seminal work by Han has demonstrated how deep learning (DL) can generate high quality sCT within seconds [13]. Following investigations further refined the DL networks and applied the method to a variety of sites and MR scanners [14]. Commercial solutions are nowadays available for head and neck, prostate and brain, while further sites such as abdomen [15], [16], [17], [18], [19] and thorax [20], [21], [22] are currently under investigation. The field is rapidly moving towards a complete coverage of all the clinically relevant scenarios but quality assurance (QA) solutions are lagging behind. The lack of uniform criteria to evaluate and report the sCT quality has been identified as one of key aspects hindering the clinical translation of novel methods [14].

The challenges in performing end-to-end tests and patient-specific QA for MR-Linac treatments have been reported [23] and additional risks have been identified when treatment planning is based on sCT without the ground truth from the planning CT [24]. National and international recommendations require an independent check of the monitor units (MU) delivered by treatment plans as a patient-specific QA (PSQA) [25], [26], [27]. Potential errors in the sCT generation propagate to the rest of the workflow [24] and a PSQA procedure for the sCT may mitigate this risk, analogously to the MU independent check. In the case of MR-only treatment planning and RT delivery at a Image-Guided Radiation Therapy (IGRT) linac, the cone-beam CT acquired just before the first fraction for patient positioning may be used to perform PSQA of the sCT [28], [29], [30]. The latter is not applicable at an MR-Linac. To overcome this, multimodality phantoms have been developed to simultaneously reproduce MR and CT contrast for generic anthropomorphic [31] and patient-specific [32] anatomies. The generation of sCT from the phantom MR scans has also been reported [32], [33]. The phantom manufacturing is however labour-intense and is therefore suited for end-to-end testing of the MR-only radiotherapy workflow rather than daily PSQA tasks. The latter may be addressed by software-based PSQA, requiring limited time allocation within the tight schedules of treatment planning for MR-Linac [34], [35], [36].

This study therefore aimed to assess software-based solutions for PSQA of synthetic CT in the context of MR-only radiotherapy at an MR-Linac.

## 2 Materials and methods

### 2.1 Patient data and sCT generation

This study retrospectively analysed 20 patients treated at the Radiation Oncology Department of the University Hospital Zurich to qualitatively assess the achievable precision of different PSQA techniques for sCT. Additionally, the data from the MR and CT simulations of a distinct group of 144 patients was used to train two neural networks. All patients were treated in the abdominal region at an hybrid MR-Linac (MRIdian v5.4.0 SN228, ViewRay, Mountain View, California, USA) [6]. All patients gave their consent for retrospective data analysis. The study was approved by the cantonal ethics committee Zurich (BASEC-Nr. 2018–01794). The former 20 patients, which were not part of the training cohort, were the main focus of the study and were selected among the treated cases to equally cover four specific sub-groups, which will be referred to as: (i) standard cases, (ii) air pockets cases, (iii) lung cases and (iv) implant cases. The first did not present air pockets or lung segments larger than 10 cubic centimetres (cc) in the axial slices including the PTV. The second and the third sub-groups were characterised by presence of air pockets or lung segments, respectively. The last group included patients with an artificial implant overlapping with the PTV. The complete patient’s characteristics, volumes of air pockets, lungs and implant type are reported in the Supplementary Table 1. Each patient underwent an MR simulation at the MR-Linac (sequence TrueFISP, field 0.35 T), followed within 30 min by a CT simulation at 120 kVp (Somatom Definition AS v64-65204 SN62425, Siemens Healthineers AG, Erlangen, Germany) in preparation for an online adaptive therapy workflow [34], [35], [36]. Step and shoot IMRT clinical plans were prepared with contouring performed on the MR and dose calculation on the deformed CT, approved by an experienced radiation oncologist and delivered. Additionally, the simulations MR were retrospectively exported to generate sCT with two in-house implementations of two separate neural networks (NN). The first was based on the pix2pix architecture [37] and was trained on a sub-set of the training cohort containing only half of the patients, similarly to the approach reported by Cusumano et al. [17]. The second was based on the CycleGAN architecture [38] and was trained on the full cohort of 144 patients, as reported by Lapaeva et al. [39]. The sCT generated by the CycleGAN model was selected as the reference sCT for dose calculation, simulating the MR-only workflow without any CT scan of the patient [40].

**Table 1:** Maximum times required to generate the dose distribution necessary to perform the quality assurance tasks. The tasks highlighted with (*) have the potential to be automated with dedicated routines handling the data flow between TPS and external software.

	(A) Water Maximum time [min]	(B) Bulk densities Maximum time [min]	Reference sCT and (C) Separate NN Maximum time [min]	(D) dCT Maximum time [min]
Export MR (*)	–	–	2 min	–
Generate CT DICOM	–	–	1 min (*)	30 min
Import DICOM (*)	–	–	2 min	2 min
Register CT-MR	–	–	–	5 min
Contour (*)	–	17.5 min	–	5 min
Generate ED map (*)	1 min	1 min	1 min	1 min
Calculate dose (*)	1 min	1 min	1 min	1 min
**Total**	**2 min**	**19.5 min**	**7 min**	**44 min**

### 2.2 Models for sCT PSQA

**Fig. 1:**
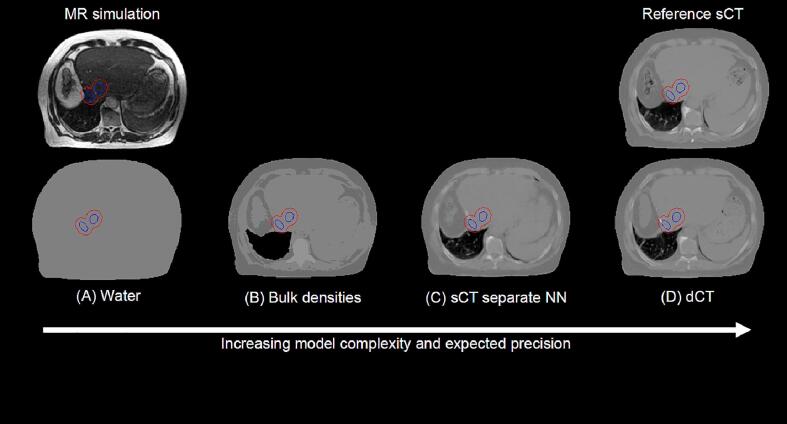
Overview of the data available in a MR-only workflow (top row) and model data (bottom row) available to perform quality assurance of the synthetic CT. The location of the PTV (red) and GTV (blue) is reported. The data shown are the MR and the electron density maps, which refers to patient number 15 in the category (iii) lung cases. (For interpretation of the references to colour in this figure legend, the reader is referred to the web version of this article.)

Four different approaches to generate separate electron density maps and perform PSQA of the CycleGAN sCT have been investigated, which will be referred to as: (A) water, (B) bulk densities, (C) separate NN and (D) dCT. The overview of the methods for an exemplary patient in the lung sub-group is shown in Fig. 1. In method A, the body mask of the patient was uniformly overridden with HU = 0. In method B, manual contouring was performed on the MR for air pockets, lung, fat, soft tissue, bone and bulk overrides of HU = -1024, −752, −148, 7 and 282 were respectively assigned. These values were the default pre-sets available in the treatment planning system (MRIdian TPS v5.3.6.11) for the corresponding tissues. In method C, the sCT generated with the pix2pix architecture was imported in the Treatment Planning System (TPS) and used to generate the electron density map. Note that the sCT generated with pix2pix and CycleGAN are referred to as separate neural networks because they are based on two different architectures and trained on either the full or a subset of the training cohort (Supplementary Table 3). Finally, method D required the import in the TPS of the planning CT, the deformable registration to the MR and the manual contouring of the air pockets, which had different spatial distribution due to the physiological changes between the MR and CT simulation, as in B to then obtain the electron density map. For the reference sCT and the cases A-D the clinically approved plan was rigidly copied to the corresponding electron density map and recalculated with the same number of monitor units using the following Monte Carlo setting: grid size 0.2 cm, magnetic field corrections activated, number of histories 2.4∙10^6^ and uncertainty 1%, corresponding to standard deviations for the Dose-Volume Histograms (DVH) points limited to less than 0.25% (Supplementary Fig. 1). This study included 100 treatment plan recalculations emerging from five methods to generate electron density maps, four patient sub-groups and five patients for each sub-group.

### 2.3 Evaluation

The time required to complete the PSQA workflow and the dose endpoint precision were evaluated. For the former, the workflow tasks from the acquisition of the MR simulation until the dose calculation were identified and the time burden measured or estimated for the 20 patients included in the PSQA study. The tasks requiring only a fraction of a minute, such as the Monte Carlo dose calculation, were rounded up to 1 min for obtaining a conservative estimation of the total workflow time. The manual contouring was performed for five tissue classes in the case of bulk densities and only for the air pockets for the deformed CT. The fat was contoured with image thresholding on the MR data. Air, bone and lung were manually contoured on the MR. The remaining voxels were assigned to soft tissue. The sCT did not require registrations because they were generated in the same frame of reference of the MR and intrinsically co-registered.

The evaluation was based on the comparison of DVH between the reference sCT and the investigated PSQA model. The DVH points were extracted for the targets (Planning Target Volume - PTV and Gross Tumor Volume - GTV Dmean, PTV D95%, PTV V95%, PTV D1cc), for a 2 cm isotropic ring surrounding the PTV (Ring 2 cm Dmean) and for the organs-at-risk (OAR). The latter included the highest near-maximum dose received by stomach, duodenum, bowel or spinal cord for a given patient (OAR D1cc). Similarly, the highest mean dose between liver, kidney and heart was extracted (OAR Dmean). The DVH dose differences between the reference sCT and the PSQA method were calculated and normalised by the prescription dose. The data processing was performed with Python v3.7.8 [41] and Digital Imaging and Communications in Medicine (DICOM) handling with the library PyMedPhys v0.38 [42].

## 3 Results

**Fig. 2:**
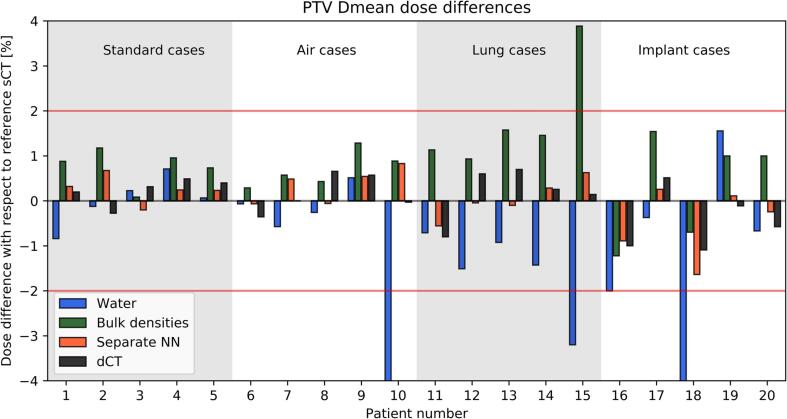
Relative signed deviation for the PTV Dmean calculated on electron density maps obtained from the reference sCT against the methods A-D. The horizontal red lines indicate the limit of ± 2%, while the vertical colour bands distinguish the patients in the four categories. (For interpretation of the references to colour in this figure legend, the reader is referred to the web version of this article.)

**Fig. 3:**
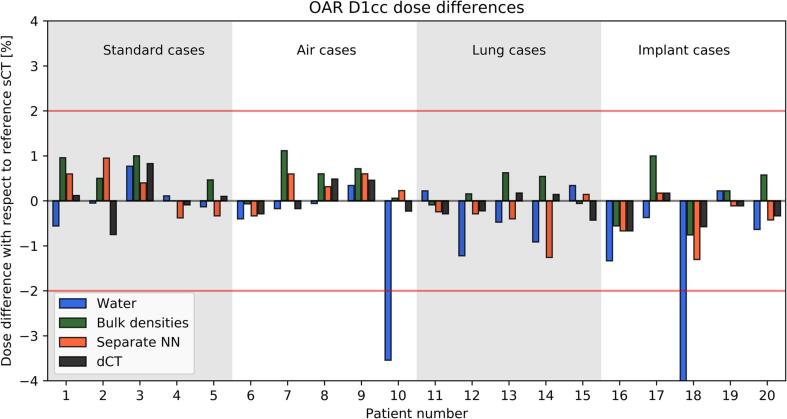
Relative signed deviation for the OAR D1cc calculated on electron density maps obtained from the reference sCT against the methods A-D. OAR D1cc corresponded to the highest near-maximum dose received by stomach, duodenum, bowel or spinal cord for a given patient. The horizontal red lines indicate the limit of ± 2%, while the vertical colour bands distinguish the patients in the four categories. (For interpretation of the references to colour in this figure legend, the reader is referred to the web version of this article.)

The processing times (Table 1) show that the methods A-C had a time burden inferior to the workflows currently implemented in clinical practice requiring a CT scan of the patient (method D). PSQA could be completed in less than 10 min for A and C, while B took up to 20 min. The DVH differences between the reference sCT and the PSQA method progressively decreased with increasing level of complexity of the model from A-D and increased with the complexity of the analysed patient subgroup from (i) to (iv). Fig. 2, Fig. 3 report the relative differences for the individual patients for the PTV Dmean and OAR D1cc, respectively. The box plots summarising the complete set of the analysed DVH points are reported in Supplementary Figs. 2–5 and individual data points are reported in Supplementary Figs. 6 and 7. The separate NN and dCT (methods C-D) were the only ones without any outlier above 2%. The bulk densities B performed well (no outliers above 3%) except for the lung cases. The dependency of the PTV Dmean on the relative electron density assigned in the bulk override is shown in the Supplementary Fig. 8 for patient Nr. 15 who was the largest outlier in Fig. 2. The CT of patient Nr. 15 demonstrated that the measured relative electron density was + 46% higher compared to the patient-independent value used in B, which if adopted in the calculation limited the differences to 3%. Furthermore, Supplementary Table 2 reports the observed signed values of the deviations for all the combinations A-D, (i)-(iv) and DVH points.

Table 2 provides a summary of the maximum observed deviations among all dose DVH points (Dmean and D1cc, excluding the volumetric DVH point V95%) for the analysed target and OAR. Differences always below 2% were observed for the separate NN and dCT (methods C-D). Bulk densities B were also within 2% except for lung (iii) and implant cases (iv). The water method A failed to provide limited deviations except for the standard cases (i).

**Table 2:** Overview of the maximum observed deviations among all the dose DVH points analysed (Dmean and D1cc) for targets and OAR structures. The deviations are reported for the combinations of PSQA methods A-D and patients subgroups (i)-(iv).

QA approach	Standard cases Maximum observed deviation [%]	Air pockets cases Maximum observed deviation [%]	Lung cases Maximum observed deviation [%]	Implant cases Maximum observed deviation [%]
(A) Water	1.0%	7.8%	3.7%	4.9%
(B) Bulk densities	1.5%	1.7%	4.6%	2.2%
(C) Separate NN	1.0%	0.9%	1.9%	1.9%
(D) dCT	1.1%	1.3%	1.8%	1.8%

**Fig. 4:**
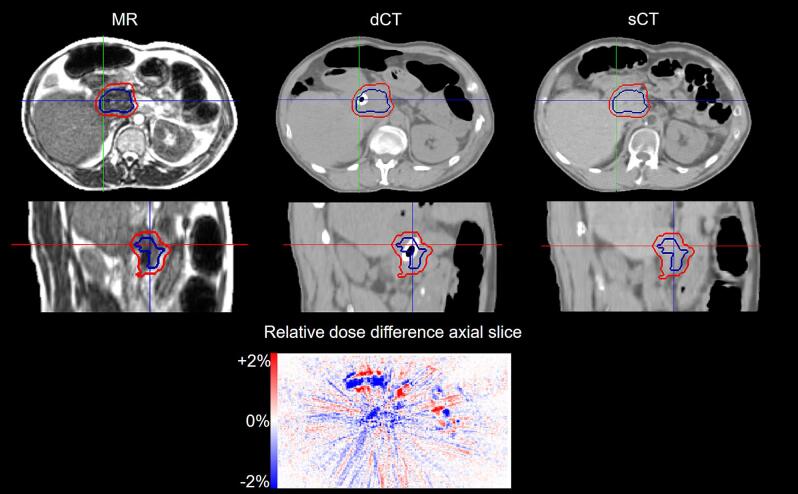
Exemplary case of sCT generation in presence of an artificial implant for patient number 18. The implant is not visible on the MR simulation (left) and not reproduced in the sCT (right). The dCT (centre) identifies the voxels with higher density. The PTV (red) and GTV (blue) contours are also reported. The relative dose difference between the reference sCT and the dCT are reported for the axial slice. (For interpretation of the references to colour in this figure legend, the reader is referred to the web version of this article.)

The artificial implants were not visible in the MR simulation and not reproduced by the PSQA methods A-C. The CT simulation identified the presence of artificial high-density material, which was reproduced in the dCT (method D). Fig. 4 reports an exemplary case in presence of a duodenal DHC metal stent. The dose differences between the reference sCT and the dCT are also reported. Pointwise underdosage up to −2% was observed in the area of the implant. For this patient subgroup (iv) the separate NN and the dCT (methods C-D) showed the largest deviations in PTV and GTV Dmean up to 1.7%, which were otherwise limited below 1% for the remaining cases (Supplementary Table 2). The OAR D1cc, OAR Dmean and Ring 2 cm Dmean were less affected, showing maximum deviations that were comparable between the implant (iv) and the other patient subgroups (i)-(iii).

## 4 Discussion

In this study we explored several possibilities for software-based PSQA of sCT, ranging from a simplistic representation of the patient with uniform water, increasing the complexity to multiple tissue classes, using a separate NN to test the reference NN and finally considering the acquisition of a backup CT. The observations suggest that the use of an additional sCT generated by a separate NN is an appropriate tool to perform PSQA of an sCT in an MR-only workflow at an MR-Linac. The time (within 10 min) and dose (within 2%) requirements were satisfied. The use of bulk density overrides may also be a valuable tool in absence of lung tissue. In case of large deviations detected simultaneously by B and C, ideally with an automated dose recalculation and comparison of a representative plan during the acquisition of additional MR sequences or directly after the MR simulation, a CT simulation of the patient may be required.

The requirements for the clinical implementation of sCT include dose deviations below 2%, or preferably better. In terms of quality assurance, the electron density map on which the dose is calculated has a direct influence on the MU assigned to the treatment beams. Therefore, approaches analogous to the independent MU calculations could be adopted. In this case, the PSQA method should also guarantee DVH equivalence within 2% or better. This study observed that a separate NN can satisfy this requirement without performing a CT scan of the patient (Table 2). The bulk density approach also respected the 2% requirement in all cases except for the implant (iv) and lung subgroup (iii). The interpretation of the latter has to be attributed to the inter-patient variability of relative electron density in the lung, which was previously reported [22], [43], [44] and confirmed in the current study (Supplementary Fig. 8).

It should be noted that the reported times (Table 1) are specific to the infrastructure employed in the current study. The total times may be significantly reduced with the introduction of automated processes within the PSQA workflow. On the other hand, the time required to acquire a CT after the MR simulation in may vary from clinic to clinic depending on the scanners’ schedules and locations within the hospital. Overall, all investigated techniques had a time burden compatible with their implementation within a clinical workflow.

The presence of artificial implants not visible in the MR simulations and not reproduced by the sCT, but also not by A-C, introduced additional variability reducing the precision of the dose calculation. In this case, the comparison with the patient CT scan is the only reliable approach to perform PSQA among the methods investigated in this study. In particular, both negative and positive deviations in the PTV Dmean were observed (Fig. 2). Nonetheless, for the separate NN, the effect on the surrounding OAR and normal tissue was limited to a maximum deviation of 1.3%, which was comparable to the deviations observed in other patient subgroups. The presence of an artificial implant is however often an exclusion criteria for the enrolment of patients into MR-only workflows [45] and it has been investigated for exploratory purposes as a worst-case scenario.

This study was limited to a specific MR-Linac system and to step and shoot IMRT photon plans. Therefore, the results cannot be generalised to other applications such as electron, photon Volumetric Modulated Arc Therapy (VMAT) or proton plans. Moreover, the separate NN employed in this study was generated by performing the training of a different model (pix2pix vs. CycleGAN) with either the full cohort or a subset of it compared to the NN for the reference sCT. The data source was a specific combination of one specific MR scanner and sequence with one CT scanner within the same clinic. A more robust approach could be based on multiple MR scanners and sequences, external data and training datasets without any overlap to achieve two completely independent NN, which however was unavailable for the current study (Supplementary Table 3). It should also be noted that this study was limited to the retrospective analysis of only 5 patients in each sub-group (i)-(iv) and therefore the maximum observed deviation was reported instead of performing statistical testing. Also, a complete PSQA protocol should verify the integrity of the data transfer between the MR scanner, TPS and NN for sCT generation, which was not within the scope of this study. Finally, the current investigation focussed primarily on the dose calculation QA for sCT, while additional QA aspects should be also taken into account: sCT to Digital Imaging and Communications in Medicine or sCT to Digitally Reconstructed Radiograph matching at an IGRT linac, end-to-end testing, imaging QA for the MR scanner and consistency of the sCT generation by the NN.

To conclude, the current study investigated the dose calculation accuracy of software-based solutions for patient specific quality assurance of synthetic CT in the context of MR-only radiotherapy at an MR-Linac. We propose the use of an additional synthetic CT generated by an independent neural network to verify the dose calculation. The verification workflow could be completed within 10 min with dose deviations within 2%.

## Declaration of Competing Interest

The authors declare the following financial interests/personal relationships which may be considered as potential competing interests: RDB was supported by the SASRO Research Grant 2022. Part of this work was supported by a research grant from ViewRay Inc. (MASPAC study), within the Clinical Research Priority Programme “Artificial intelligence in Oncological Imaging'' of the University of Zurich, and SNF R’Equip program (grant 326030_177080/1).
