# Supplementary material for: Patient-specific quality assurance strategies for synthetic computed tomography in magnetic resonance-only radiotherapy of the abdomen
Source: Phys Imaging Radiat Oncol. 2023 Jun 26;27:100464. doi: 10.1016/j.phro.2023.100464 (PMC10366576; doi:10.1016/j.phro.2023.100464)
Supplement: Supplementary data 1 [file mmc1.docx]

**Supplementary materials**

| **Patient** | **Class** | **Prescription** | **ICD-10** | **Notes** |
| --- | --- | --- | --- | --- |
| 1 | Standard | 5x5Gy @80% | C77.2 | V(air) < 10 cc and V(lung) < 10 cc |
| 2 | Standard | 5x8Gy @65% | C78.7 | V(air) < 10 cc and V(lung) < 10 cc |
| 3 | Standard | 5x7Gy @65% | C79.7 | V(air) < 10 cc and V(lung) < 10 cc |
| 4 | Standard | 5x9Gy @65% | C78.7 | V(air) < 10 cc and V(lung) < 10 cc |
| 5 | Standard | 5x6Gy @65% | C78.6 | V(air) < 10 cc and V(lung) < 10 cc |
| 6 | Air | 5x9Gy @65% | C78.7 | V(air) = 46.42 cc |
| 7 | Air | 5x7Gy @65% | C25.1 | V(air) = 191.61 cc |
| 8 | Air | 5x7Gy @80% | C79.0 | V(air) = 17.26 cc |
| 9 | Air | 5x7Gy @80% | C25.9 | V(air) = 48.91 cc |
| 10 | Air | 10x3.5Gy @80% | C79.7 | V(air) = 333.96 cc |
| 11 | Lung | 5x9Gy @65% | C78.7 | V(lung) = 318.05 cc  Lung in N=21 slices with PTV |
| 12 | Lung | 5x9Gy @65% | C78.7 | V(lung) = 640.13 cc  Lung in N=15 slices with PTV |
| 13 | Lung | 5x8Gy @65% | C78.7 | V(lung) = 478.81 cc  Lung in N=19 slices with PTV |
| 14 | Lung | 5x7Gy @65% | C78.7 | V(lung) = 1012.20 cc  Lung in N=25 slices with PTV |
| 15 | Lung | 5x7Gy @65% | C78.7 | V(lung) = 549.67 cc  Lung in N=16 slices with PTV |
| 16 | Implant | 3x1.8Gy (boost) | C24.9 | Duodenal DHC metal stent |
| 17 | Implant | 5x7Gy @65% | C79.9 | Roux-y gastric bypass |
| 18 | Implant | 5x6.6Gy @80% | C25.0 | Duodenal DHC metal stent |
| 19 | Implant | 3x3Gy (boost) | C24.0 | Pancreatic and biliary metal stent |
| 20 | Implant | 5x6.6Gy @80% | C25.0 | Pancreatic and biliary metal stent |

Supplementary Table 1. Patient characteristics and notes affecting the dose calculation. The volume of air and lung was evaluated on the MR for the axial slices including the PTV and additional 2 cm in cranial and caudal direction. The implants had an overlap with the PTV.

| Parameter | QA approach | Standard cases  Mean (range) [%] | Air pockets cases  Mean (range) [%] | Lung cases  Mean (range) [%] | Implant cases  Mean (range) [%] |
| --- | --- | --- | --- | --- | --- |
| PTV Dmean | Water | 0.0 (-0.8 - 0.7) | **-1.3 (-6.2 - 0.5)** | **-1.6 (-3.2 - -0.7)** | **-1.1 (-4.1 - 1.6)** |
|  | Bulk densities | 0.8 (0.1 - 1.2) | 0.7 (0.3 - 1.3) | **1.8 (0.9 - 3.9)** | 0.3 (-1.2 - 1.5) |
|  | Separate NN | 0.3 (-0.2 - 0.7) | 0.4 (-0.1 - 0.8) | 0.0 (-0.6 - 0.6) | -0.5 (-1.6 - 0.3) |
|  | dCT | 0.2 (-0.3 - 0.5) | 0.2 (-0.4 - 0.7) | 0.2 (-0.8 - 0.7) | -0.5 (-1.1 - 0.5) |
| PTV D95% | Water | 0.0 (-0.8 - 0.5) | **-1.6 (-6.8 - 0.1)** | **-0.8 (-2.1 - 0.1)** | **-1.0 (-3.7 - 1.2)** |
|  | Bulk densities | 0.6 (0.3 - 0.8) | 0.4 (-0.6 - 1.1) | **1.5 (1.0 - 2.8)** | 0.3 (-1.0 - 1.1) |
|  | Separate NN | 0.3 (0.0 – 1.0) | 0.0 (-0.2 - 0.5) | 0.2 (-0.1 - 0.6) | -0.3 (-0.9 - 0.3) |
|  | dCT | 0.1 (-0.8 - 0.7) | -0.1 (-1.3 - 0.5) | 0.4 (-0.2 - 1.1) | -0.2 (-0.6 - 0.2) |
| PTV V95% | Water | **-0.4 (-2.8 - 0.3)** | **-2.1 (-10.5 - 0.7)** | -0.6 (-1.0 - -0.3) | **-1.3 (-6.4 - 2.3)** |
|  | Bulk densities | **1.0 (0.2 - 2.9)** | 0.6 (-0.4 - 1.8) | 0.9 (0.1 – 2.0) | 0.3 (-1.7 - 1.8) |
|  | Separate NN | 0.4 (-0.1 - 1.3) | 0.2 (-0.2 - 0.7) | 0.1 (-0.3 - 0.3) | -0.7 (-2.1 - 0.2) |
|  | dCT | 0.3 (0.1 - 0.6) | 0.1 (-0.9 - 0.9) | 0.1 (-0.3 - 0.5) | -0.6 (-1.3 - 0.4) |
| PTV D1cc | Water | -0.1 (-1.0 - 0.8) | **-1.6 (-7.8 – 1.0)** | **-1.7 (-3.6 - -0.3)** | **-1.2 (-4.6 - 1.8)** |
|  | Bulk densities | 0.9 (-0.1 - 1.4) | 0.6 (-0.2 - 1.7) | **2.1 (0.8 - 4.6)** | **0.3 (-1.6 - 2.2)** |
|  | Separate NN | 0.2 (-0.5 - 0.6) | 0.1 (-0.5 - 0.7) | 0.4 (-0.6 - 1.9) | -0.5 (-1.9 - 0.8) |
|  | dCT | 0.0 (-0.6 - 0.4) | 0.0 (-1.2 - 0.8) | 0.2 (-1.2 - 1.8) | -0.6 (-1.8 – 1.0) |
| GTV Dmean | Water | 0.0 (-1.0 - 0.9) | **-1.4 (-6.5 - 0.7)** | **-2.1 (-3.7 - -0.7)** | **-1.2 (-4.4 - 1.6)** |
|  | Bulk densities | 0.8 (0.0 - 1.3) | 0.8 (0.4 - 1.4) | **1.7 (0.2 - 4.4)** | 0.3 (-1.4 - 1.8) |
|  | Separate NN | 0.3 (-0.4 - 0.8) | 0.4 (-0.1 - 0.7) | -0.1 (-0.6 - 0.7) | -0.6 (-1.7 - 0.3) |
|  | dCT | 0.4 (-0.3 - 1.1) | 0.2 (-0.2 - 0.7) | 0.1 (-1.0 - 0.7) | -0.5 (-1.4 - 0.7) |
| Ring 2cm Dmean | Water | 0.1 (-0.3 - 0.2) | **-0.4 (-2.0 - 0.3)** | **-1.0 (-1.4 - -0.6)** | -0.4 (-1.3 - 0.7) |
|  | Bulk densities | 0.3 (0.1 - 0.5) | 0.3 (0.1 - 0.4) | 0.7 (0.6 - 1.3) | 0.3 (-0.1 - 0.6) |
|  | Separate NN | 0.1 (0.0 - 0.3) | 0.1 (-0.1 - 0.3) | -0.1 (-0.4 - 0.3) | -0.3 (-0.9 - 0.1) |
|  | dCT | 0.1 (0.1 - 0.2) | 0.1 (-0.2 - 0.2) | 0.1 (-0.2 - 0.4) | -0.1 (-0.3 - 0.2) |
| OAR D1cc | Water | 0.0 (-0.6 - 0.8) | **-0.8 (-3.5 - 0.3)** | -0.4 (-1.2 - 0.3) | **-1.4 (-4.9 - 0.2)** |
|  | Bulk densities | 0.6 (0 - 1) | 0.5 (-0.1 - 1.1) | 0.2 (-0.1 - 0.6) | 0.1 (-0.8 – 1.0) |
|  | Separate NN | 0.3 (-0.4 – 1.0) | 0.3 (-0.3 - 0.6) | -0.4 (-1.3 - 0.1) | -0.5 (-1.3 - 0.2) |
|  | dCT | 0.0 (-0.8 - 0.8) | 0.1 (-0.3 - 0.5) | -0.1 (-0.4 - 0.2) | -0.3 (-0.7 - 0.2) |
| OAR Dmean | Water | 0.1 (0.0 - 0.3) | 0.0 (-0.2 - 0.2) | -0.0 (-0.5 - 0.1) | 0.3 (-0.2 - 1.2) |
|  | Bulk densities | 0.4 (0.1 - 1.5) | 0.2 (0.1 - 0.2) | 0.5 (0.3 - 0.7) | 0.5 (0.1 - 1.3) |
|  | Separate NN | 0.0 (0.0 - 0.1) | 0.1 (0.0 - 0.1) | 0.0 (-0.1 - 0.2) | 0.2 (-0.1 - 0.9) |
|  | dCT | 0.1 (0.0 - 0.3) | 0.1 (0.0 - 0.2) | 0.1 (-0.1 - 0.3) | 0.3 (0.0 - 0.9) |

Supplementary Table 2. Quantitative values of the dosimetric differences for the DVH dosimetric points between the calculation performed on the reference sCT against the methods A-D. Entries exceeding 1% in mean or 2% in range are highlighted in bold.

| Independent properties | Common properties |
| --- | --- |
| 1. 72 patients present only in the CycleGAN training cohort 2. Neural network architecture 3. Number of patients in the training set 4. Paired images vs unpaired images training | 1. 72 patients present in both the pix2pix and CycleGAN training cohort 2. MR and CT scanners used to acquire data 3. MR sequence used to acquire data 4. Patient data from the same clinic |

Supplementary Table 3. Overview of the independent and common properties for the neural networks used to generate the reference sCT for dose calculation (CycleGAN) and for method C (pix2pix).


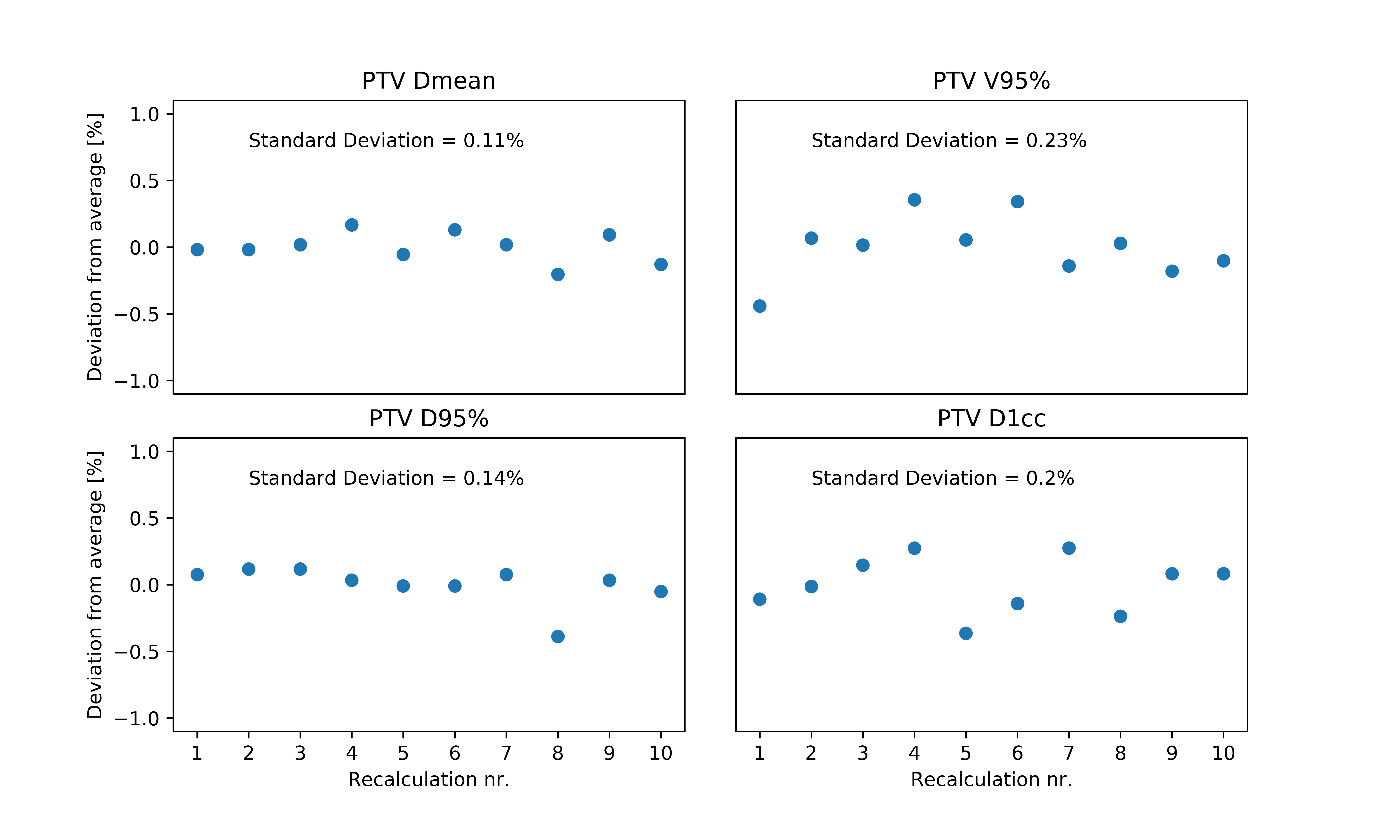


Supplementary Figure 1. Evaluation of the uncertainty due to the Monte Carlo calculation. The data refers to ten consecutive recalculations for Patient 01. Representative parameters for the PTV including mean dose, coverage and near maximum are reported along with the standard deviation.


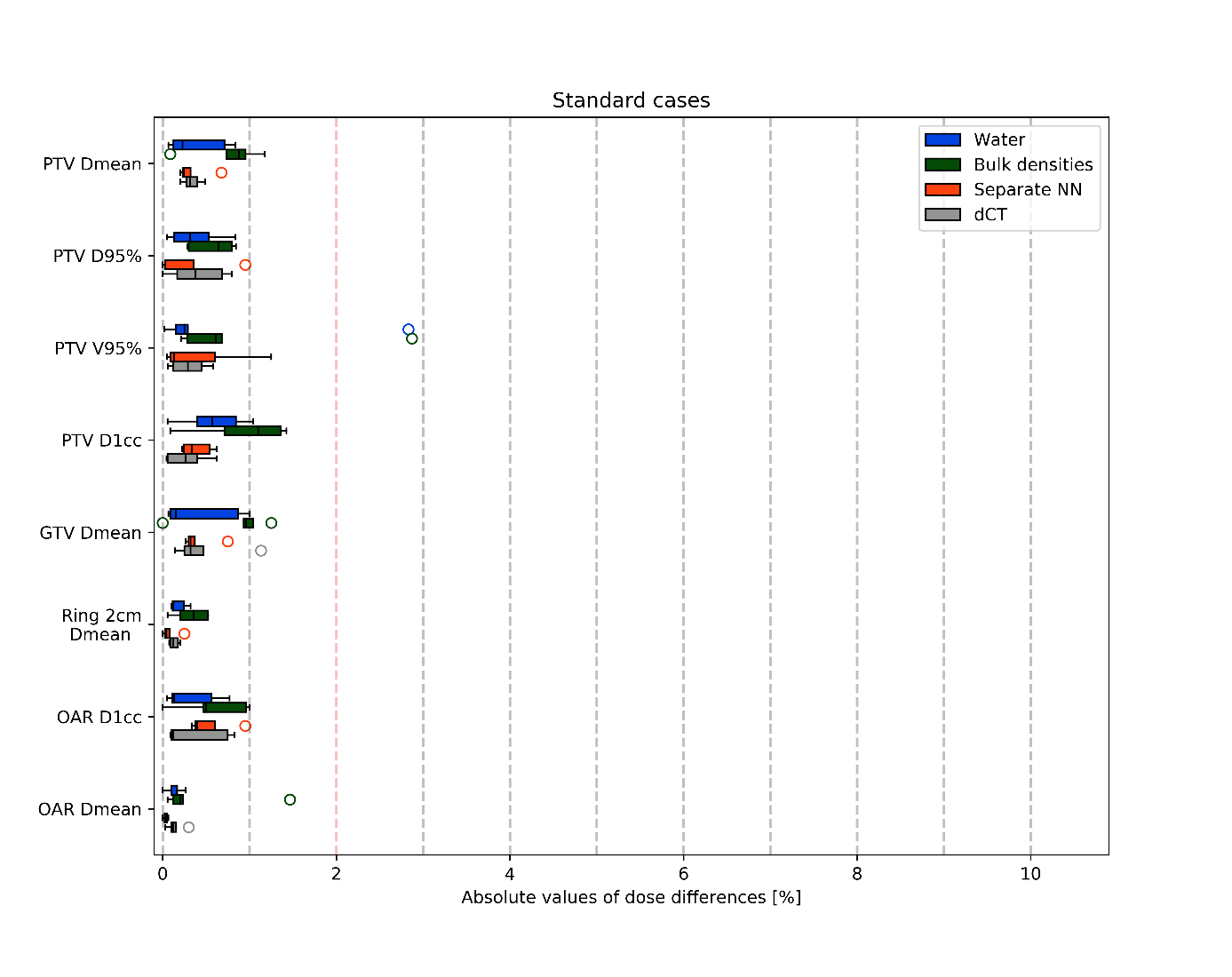


Supplementary Figure 2. Boxplots of the absolute values of the relative differences for the DVH dosimetric points calculated on the electron density maps obtained from the reference sCT against the methods A-D. The figure includes the data from the patient sub-group (i). The vertical red line represents the 2% limit for the deviations.


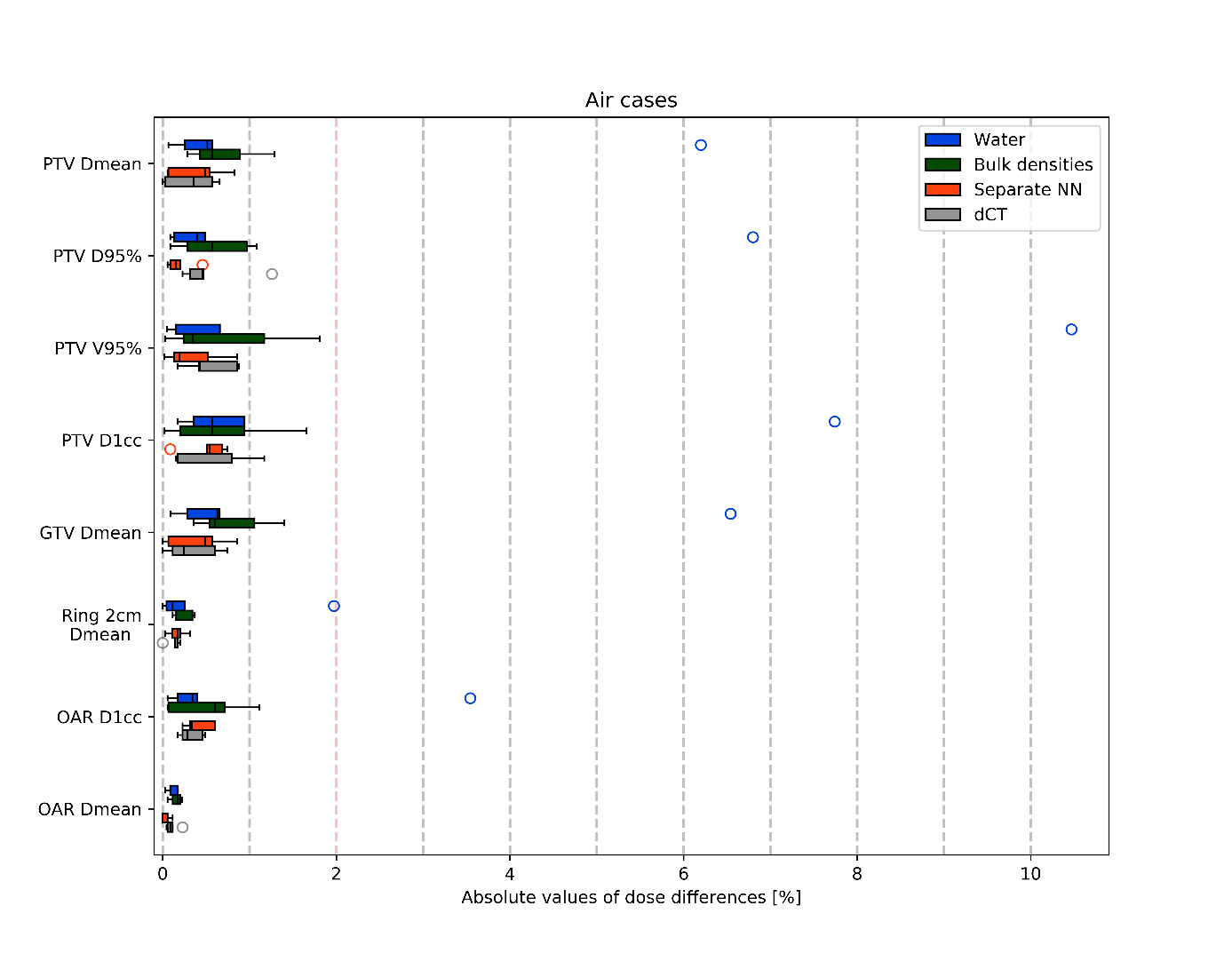


Supplementary Figure 3. Boxplots of the absolute values of the relative differences for the DVH dosimetric points calculated on the electron density maps obtained from the reference sCT against the methods A-D. The figure includes the data from the patient sub-group (ii). The vertical red line represents the 2% limit for the deviations.


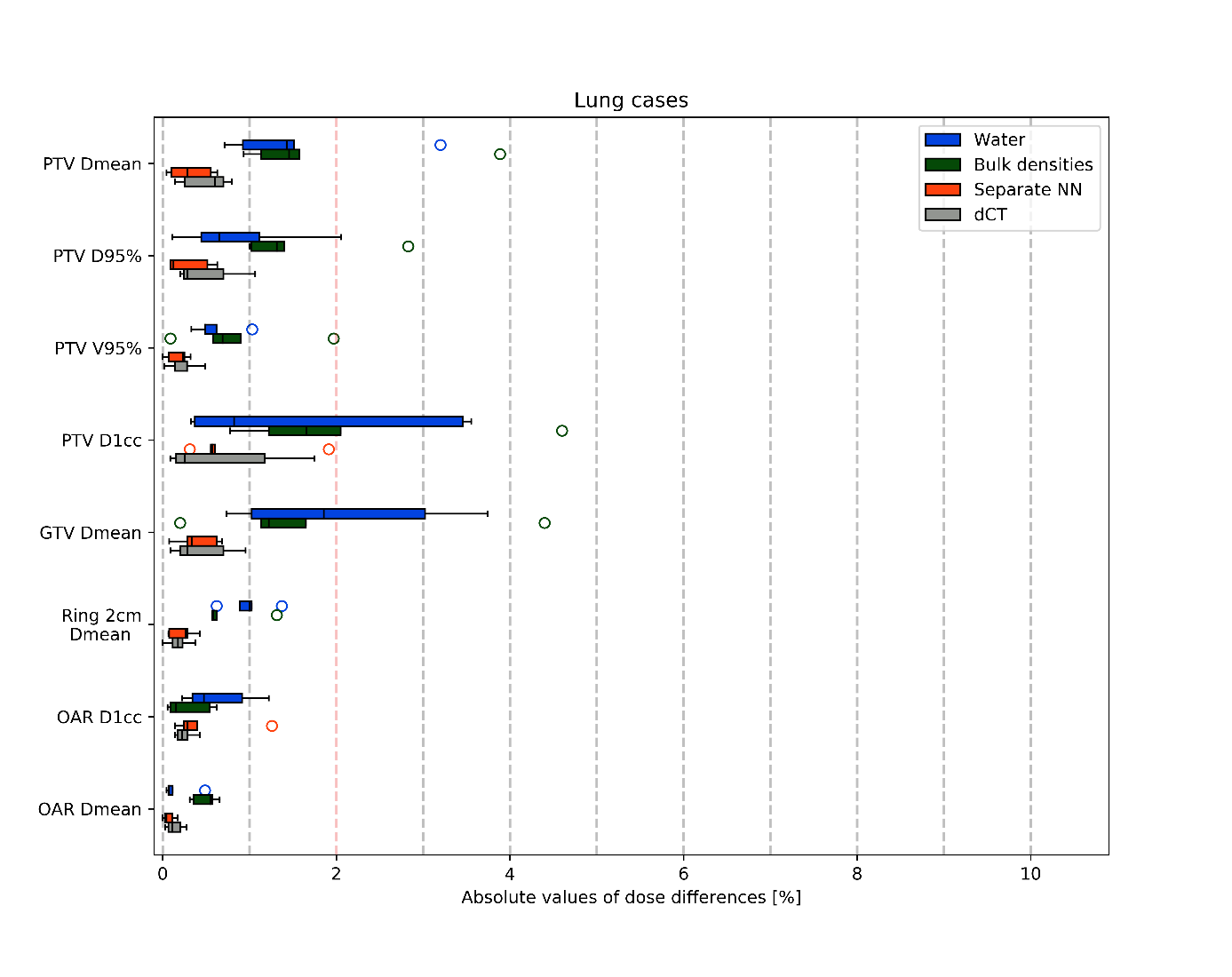


Supplementary Figure 4. Boxplots of the absolute values of the relative differences for the DVH dosimetric points calculated on the electron density maps obtained from the reference sCT against the methods A-D. The figure includes the data from the patient sub-group (iii). The vertical red line represents the 2% limit for the deviations.


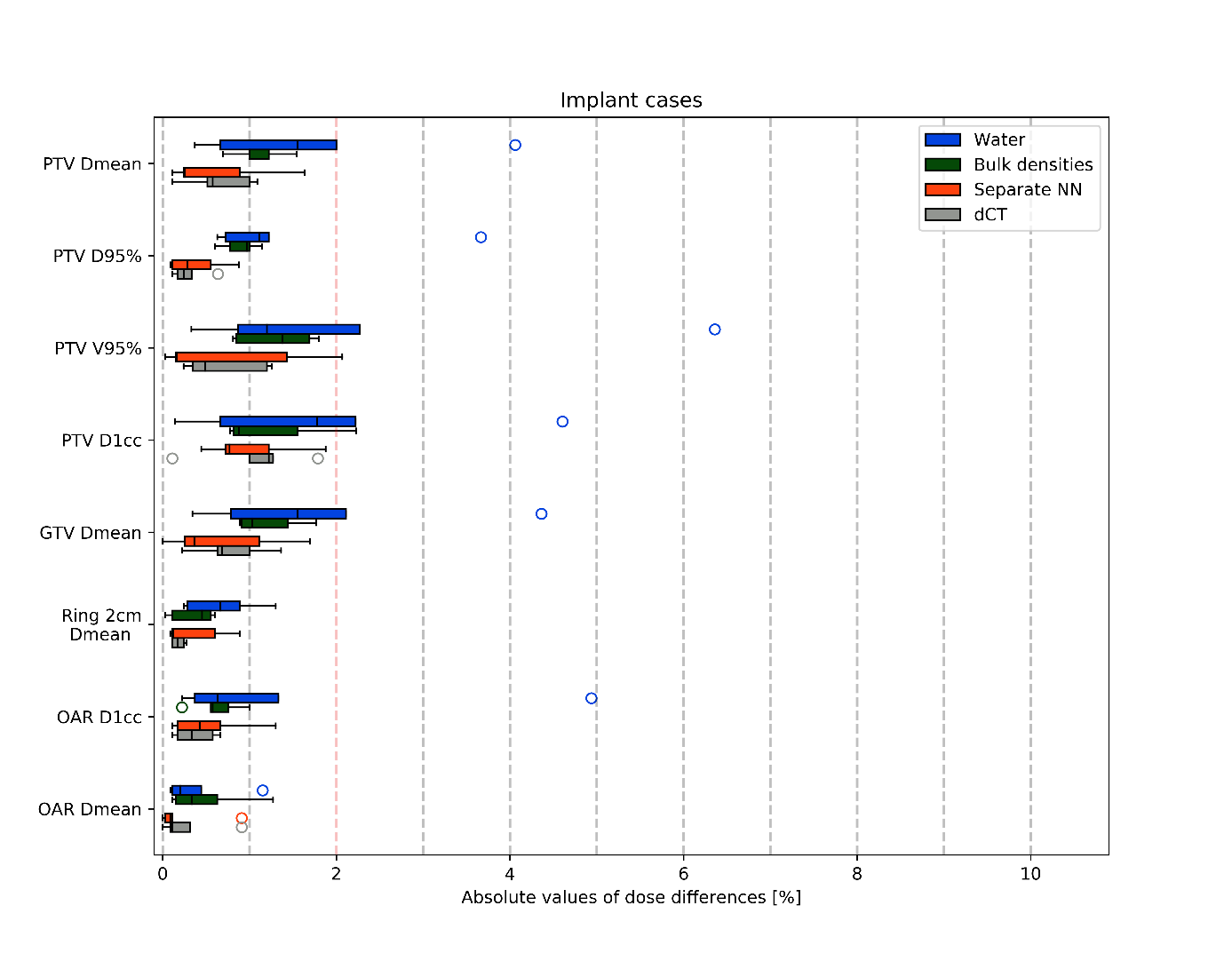


Supplementary Figure 5. Boxplots of the absolute values of the relative differences for the DVH dosimetric points calculated on the electron density maps obtained from the reference sCT against the methods A-D. The figure includes the data from the patient sub-group (iv). The vertical red line represents the 2% limit for the deviations.


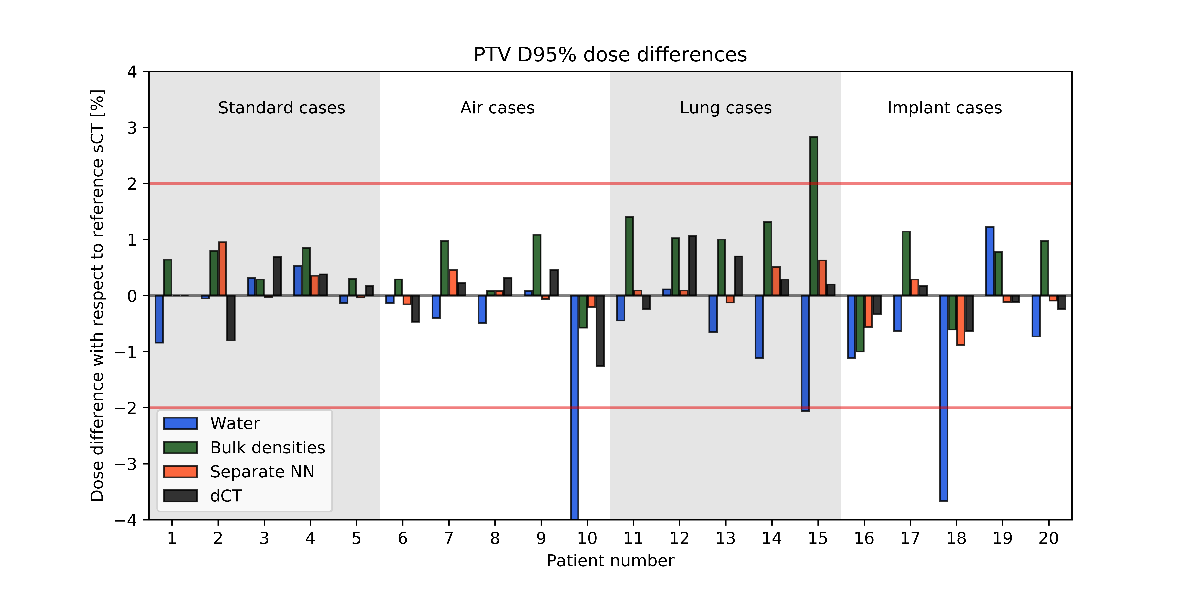


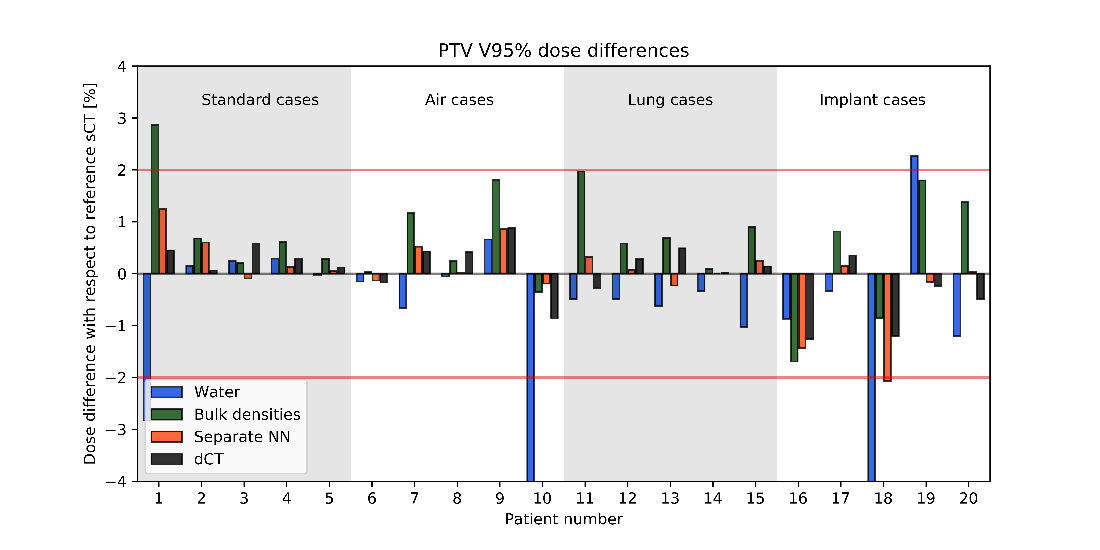

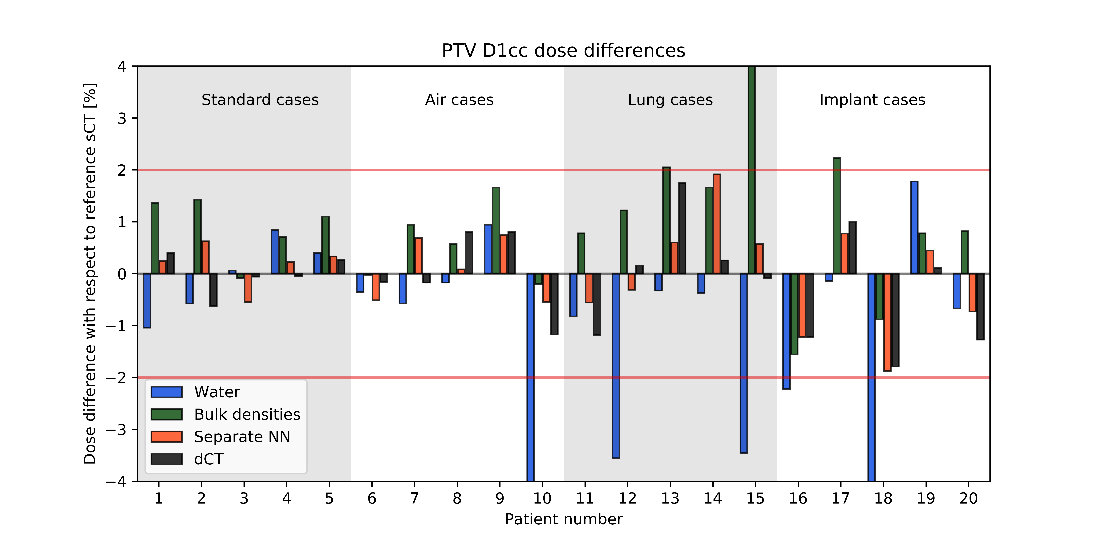


Supplementary Figure 6. Relative signed deviation for the PTV D95% (top), PTV V95% (middle) and PTV D1cc (bottom) calculated on electron density maps obtained from the reference sCT against the methods A-D. The horizontal red lines indicate the limit of ±2%, while the vertical colour bands distinguish the patients in the four categories.


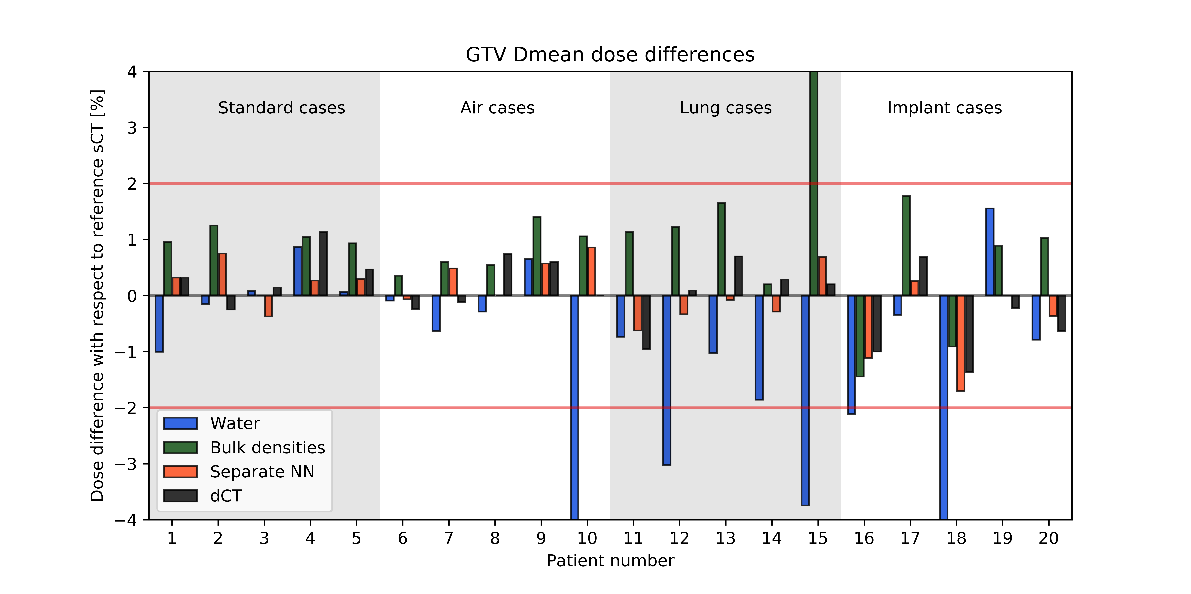

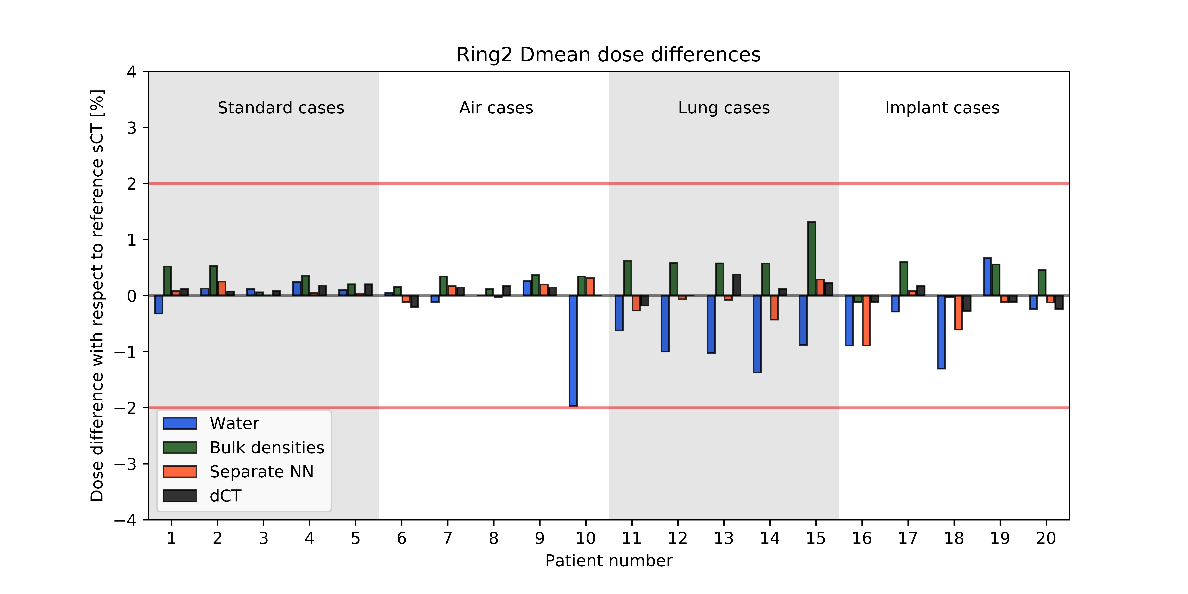

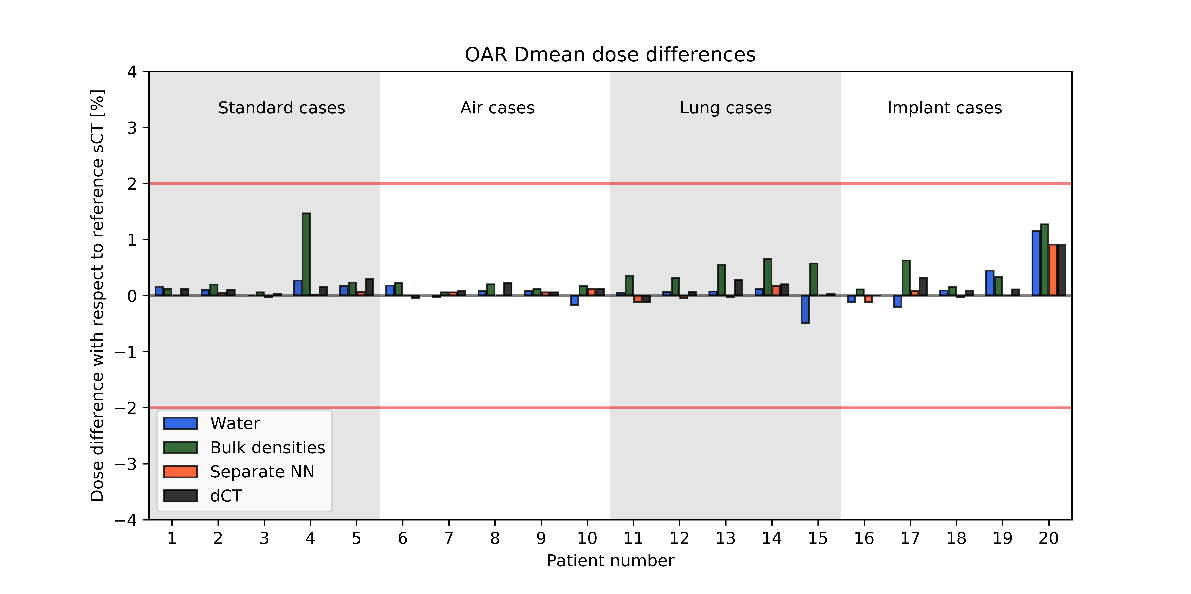


Supplementary Figure 7. Relative signed deviation for the GTV Dmean (top), Ring2 Dmean (middle) and OAR Dmean (bottom) calculated on electron density maps obtained from the reference sCT against the methods A-D. The horizontal red lines indicate the limit of ±2%, while the vertical colour bands distinguish the patients in the four categories.


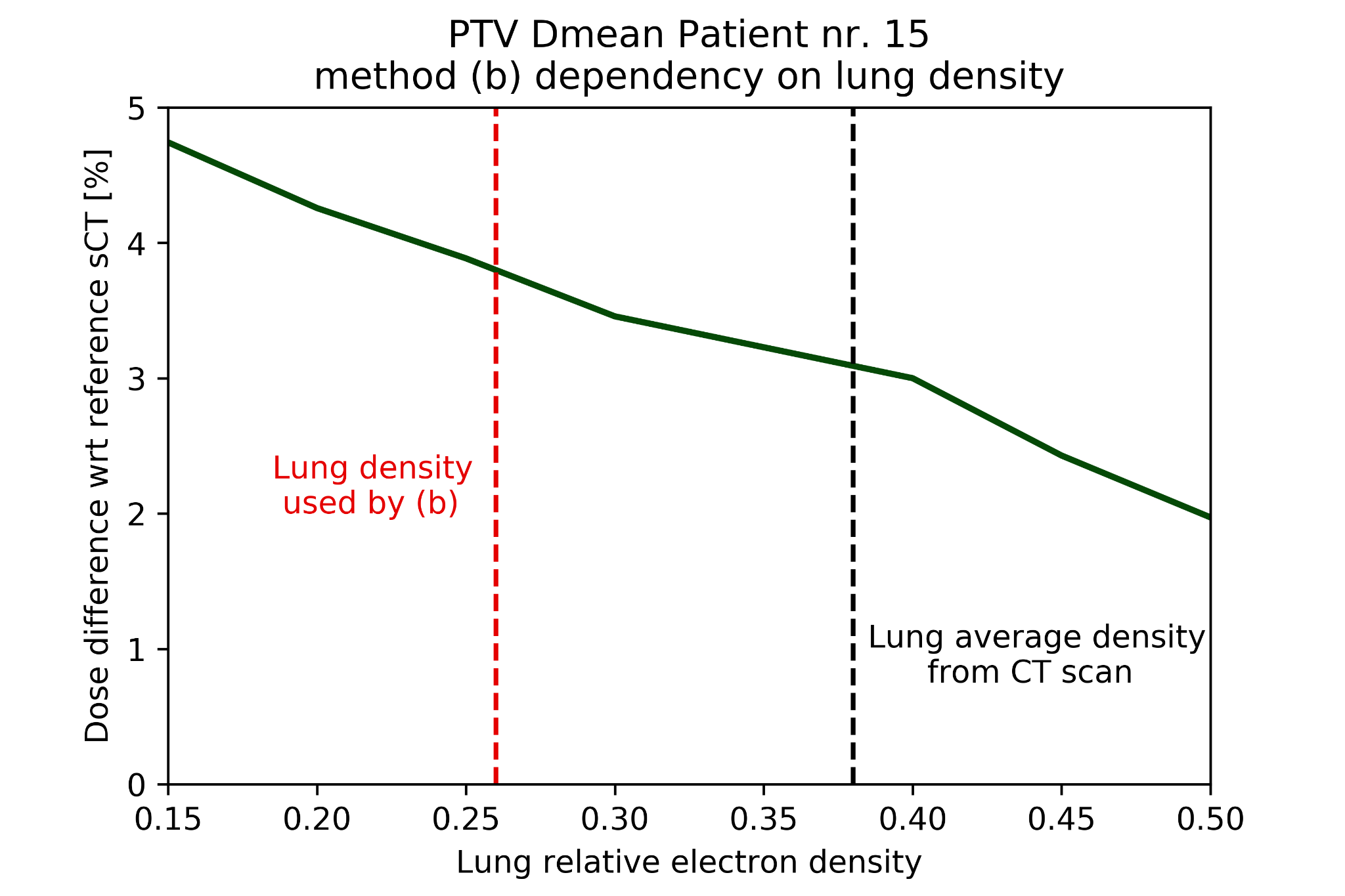


Supplementary Figure 8. Dependency of the PTV Dmean dose difference on the assigned lung electron density for the method B and the patient number 15. The vertical dashed lines indicate the bulk density adopted the method B and the average density over the distribution of the lung voxels in method D, which were respectively 0.26 and 0.38.
